# Linking Fluorine with Bio-Derived Furfural: Aiming Towards More Sustainable Fluorinated Polymers and Drugs

**DOI:** 10.3390/molecules30112305

**Published:** 2025-05-24

**Authors:** Konstantin I. Galkin, Irina V. Sandulenko

**Affiliations:** 1 N. D. Zelinsky Institute of Organic Chemistry, Russian Academy of Sciences, Leninsky Prospekt, 47, Moscow 119991, Russia; 2 Center for Project Activities, Moscow Polytechnic University (Moscow Polytech), Bolshaya Semyonovskaya Str., 38, Moscow 107023, Russia; 3 A. N. Nesmeyanov Institute of Organoelement Compounds, Russian Academy of Sciences, Vavilova Street, 28, Moscow 119991, Russia; ira17.rock@mail.ru

**Keywords:** furfural, fluorination, fluoroalkylation, pharmaceuticals, fluoropolymers

## Abstract

This perspective highlights current trends and recent advances in the introduction of fluorine and fluoroalkyl moieties into the furanic core of biobased furfural-derived furans. Existing and potential applications of these fluorinated building blocks in the development of pharmaceuticals and advanced materials are also discussed.

## 1. Introduction

While naturally occurring organofluorine compounds are rare, [1] these synthetic products have a significant impact on academic and industrial fields. Fluorine is the most electronegative element that is sterically similar to hydrogen [2]; therefore, carbon–fluorine bonds are characterized by high stability, low polarization and a strong electron-withdrawing ability [3]. Consequently, the introduction of a fluorine atom does not substantially increase the volume of the molecule, yet it often leads to significant changes in physical, chemical, stereochemical, physicochemical, and biological properties of small organic molecules and polymers [4,5,6,7,8].

Due to their unique advantages, fluorinated compounds are widely used, particularly in the development of advanced pharmaceuticals, agrochemicals, and materials [9,10,11,12]. The importance of fluorine in the development of pharmaceuticals and agrochemicals is mostly a consequence of the considerable influence of fluorination on bioactivity profiles, metabolic stability, and physicochemical properties such as acidity/basicity, lipophilicity, and solubility [2,5,13,14,15,16,17]. Additionally, fluorine atoms can participate in hydrogen bonding as electron pair donors, thereby stabilizing certain conformations [18]. These advantages of fluorination contribute to fluorine-containing compounds accounting for approximately 20–25% of approved small molecule pharmaceuticals, with 30–40% of agrochemicals containing at least one fluorine atom in their structure [19,20,21,22,23,24,25]. Numerous fluorinated derivatives with notable biological activity have been synthesized, including indoles [26,27], quinazolines [28,29], steroids [30], and morphinans [31,32,33].

Notably, the trifluoromethyl group is the most commonly used fluorinated group [34]. In biological studies and materials, it is particularly attractive due to the greater chemical stability of trifluoromethylated products compared to difluoromethyl- and monofluoromethyl-containing analogues, as well as its relatively low toxicity [35]. The steric demand of a trifluoromethyl group is comparable to that of an isopropyl group, and its electronegativity is similar to that of oxygen [18]. Incorporating trifluoromethyl groups into organic compounds can significantly modify their chemical reactivity, acidity, polarity, lipophilicity, metabolic stability, and binding selectivity [34,36].

Direct trifluoromethylation with or without transition metal catalysts offers an important pathway for synthesizing fluorinated targets, but it often suffers from poor selectivity, low yields, harsh reaction conditions, and the usage of corrosive and expensive CF_3_ sources, which restricts the feasibility of this approach [37,38,39]. Over the past decade, substantial efforts have been made to address these limitations. Consequently, numerous efficient methods for the direct incorporation of trifluoromethyl groups into heterocyclic molecules have been developed, utilizing electrophilic, nucleophilic, and radical trifluoromethylating reagents [34,40,41,42]. Compared to nucleophilic and electrophilic trifluoromethylation, radical trifluoromethylation allows for the direct introduction of CF_3_ into organic molecules without the need for additional steps to prepare functionalized substrates (such as organohalides and organometallic reagents), thereby providing a more efficient route to access trifluoromethylated compounds [43,44].

Carbo- and heterocycles containing fluorine or fluoroalkyl moieties are common structural motifs of many bioactive substances [9,20,45]. On the other hand, furans are perhaps one of the most prominent classes of heteroaromatic compounds with widespread occurrence in nature [46]. Numerous bioactive natural and synthetic products such as pharmaceuticals, agrochemicals and flavouring agents contain a furan ring in their structure [47,48,49,50,51,52]. The insufficient stability of many furanic compounds significantly limits their synthetic utility [53,54,55]. The presence of strong electron-withdrawing F-containing substituents at the α-carbon (C2 or C5) positions markedly improves the furan ring stability under acidic conditions [56,57]. Consequently, fluorine-functionalized furans have emerged as particularly valuable scaffolds in fine organic synthesis and prospective biologically active compounds [56,57].

**Figure 1:**
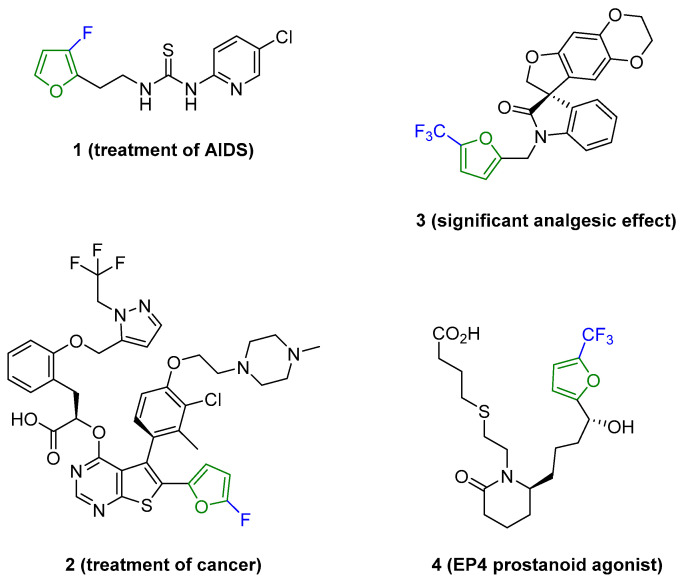
Representative examples of bioactive compounds with fluorofuran and trifluoromethylfuran functionalities. Color indicates the fluorine-functionalized furan fragment.

Fluorine-containing furans have been utilized in the design of drugs for treatment of various diseases [57,58,59,60,61,62,63]. For instance, β-fluorofuran derivative **1**, a structural analogue of trovirdine (LY 300046), exhibits high amounts of activity against the human immunodeficiency viruses (HIV) (Figure 1) [64]. Compound **2** with a α-fluorofuran moiety is a potent and selective inhibitor of the induced myeloid leukemia cell differentiation protein MCL1 and is considered a promising candidate for targeted cancer therapy [65]. Trifluoromethylated furan subunits can often be found in novel structures for drug development and agrochemicals (examples of biologically active α-trifluoromethylated furans **3** and **4** are presented on Figure 1) [21,23,60,66]; they have also found prospective applications in various materials including liquid crystals, photoresist polymers, and self-assembling monolayers [60].

Fluorinated and fluoroalkylated furans may serve as valuable synthetic intermediates. While numerous methods for the nucleophilic or transition-metal-catalyzed defluorinative C−F bond functionalization of fluorinated heterocycles are reported [67,68,69], information regarding the application of these methods for modifying fluorine-functionalized furans is limited [70]. On the other hand, the high electronegativity of the fluorine atom can make the adjacent C–H bond in a furan ring more susceptible to nucleophilic attacks that may facilitate the transformation of fluorine-containing furans into more complex fluorinated structures [71,72]. Furthermore, fluorofurans and fluoroalkylfurans can be utilized as building blocks for synthesizing non-furanic cyclic or acyclic compounds [73].

Furfural is a key renewable furanic compound produced industrially from plant biomass [74,75,76]. Due to its availability and diverse reactivity, furfural has been selected as a platform chemical [77] and is actively used as a renewable building block for biofuels, fine chemicals, monomers, and polymers [78,79,80,81]. One of the actual and promising areas of application of furfural and other bioderived furans is the sustainable production of pharmaceuticals and other bioactive compounds [82,83].

In contrast to traditional aromatic polymers, furfural-derived polymers enable the transformation into three-dimensional dynamic thermosets by utilizing the reactivity of the furan ring in Diels–Alder reactions with maleimides [84,85]. Dynamic thermosets, also known as covalent adaptable networks (CANs), dynamers or vitrimers, combine the advantages of traditional thermosets such as heat, chemical, and creep resistance, high mechanical strength, and electrical insulation with the ability to be reprocessed like thermoplastics through the activation of dynamic covalent bonds [86,87]. Current research on furan–maleimide Diels–Alder (FMDA) thermosets focuses on developing sustainable and biobased smart materials, which combine excellent mechanical properties with effective self-healing efficiency and recycling capabilities [88,89]. The self-healing mechanism in FMDA polymers relies on the reversibility of the furan–maleimide Diels–Alder reaction, which can be initiated by various external stimuli such as temperature, light, and mechanical or magnetic forces [84,85].

**Scheme 1:**
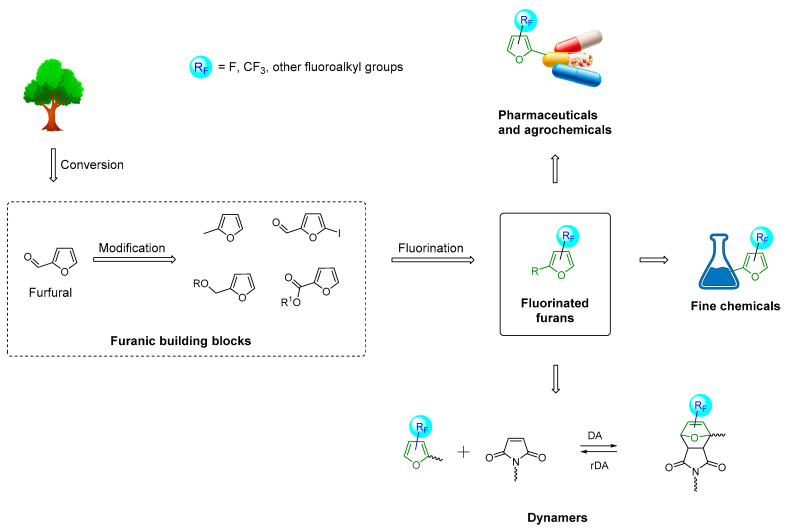
Synthetic pathways for fluorinated fine chemicals, bioactive compounds, and dynamic polymers using furfural as a renewable building block.

The modification of furfural into fluorinated furanic building blocks presents an intriguing approach to linking the biomass conversion with the production of fluorinated fine chemicals, advanced dynamic polymers, and pharmaceuticals (Scheme 1). Application of biobased furans as starting materials makes the production of targeted fluorinated products more sustainable and environmentally friendly in agreement with modern trends towards green chemical industry. In this perspective, we analyze the current challenges and recent advancements in introducing fluorine or fluoroalkyl functionalities into the furanic core of biobased furfural and most important derivatives. We also discuss the progress in applications of these fluorinated furanic building blocks in the development of pharmaceuticals and dynamic polymers.

## 2. Fluorination and Fluoroalkylation of Furfural-Derived Furans

While numerous methods for the incorporation of fluorine or fluorine-containing functional groups into various aromatic and heteroaromatic structures have been reported, [10,34,41,90,91,92], the functionalization of furanic building blocks remains underdeveloped. The scientific literature includes a limited number of reviews focused on the synthesis of fluorinated furans, with the most comprehensive reviews addressing the advances in the preparation of fluorofuran and fluoroalkylfuran derivatives covering the corresponding publications only up to 2015 [93,94]. In this paper, we discuss studies on the synthesis of fluorofuran and fluoroalkylfuran building blocks, primarily published over the last decade.

The carbon–fluorine bond formation is a challenging chemical transformation [37]. Two fundamentally distinct strategies exist for introducing fluorine-containing functional groups into furans and other heterocycles: (1) direct substitution of a hydrogen or functional group in the heterocyclic ring with fluorinated reagents or (2) cyclocondensation/cycloaddition reactions with fluorinated acyclic building blocks [18,56,66,95]. The direct introduction of fluorine and various fluoroalkyl functional groups into heteroaromatic cores can be performed with or without a catalyst through electrophilic, nucleophilic, or radical mechanisms [37,43,96,97,98,99,100]. Various environmental approaches such as the use of organocatalysts [101], electrocatalysis [40], or in-water fluorination [102] are also actively explored. The introduction of fluorinated functional groups via C–H activation eliminates the requirement for pre-functionalizing the substrate. This also enables selective functionalization at a late stage in the synthesis, which is particularly advantageous when developing new drugs and other high-value chemical products [99,103].

**Scheme 2:**
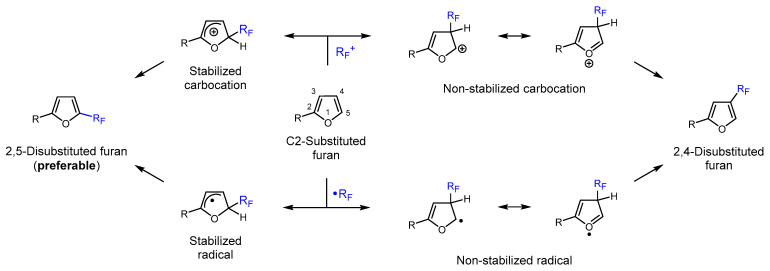
Possible pathways for introduction of fluorinated functional groups into 2-substituted furan derivatives by electrophilic or radical mechanisms. R_F_^+^ = fluorinated electrophile, •R_F_ = fluorinated radical. Here and below, blue color indicates fluorine or a fluorinated functional group.

While approaches for the direct introduction of fluorinated functional groups into heterocycles look preferable and conceptually simpler than the use of fluorinated acyclic precursors, these methods often face challenges [18,56,57,66]. Despite the longstanding appreciation of fluorine utility, many fluorination methods still lack generality, practicality, and predictability [37,103]. Moreover, most fluorinating agents are costly, toxic, corrosive, and potentially explosive or exhibit too high reactivity, which can lead to undesired modifications in the existing functional groups [36,57,104,105,106]. Due to the electron-rich nature of the furan ring, the direct introduction of fluorinated functional groups into the furanic core predominantly occurs through electrophilic or radical substitution [93,94]. This functionalization of 2-substituted furans typically takes place selectively at the C5 position, owing to the greater stabilization of the corresponding carbocations and radicals (Scheme 2) [76,94,107,108].

### 2.1. Synthesis of Fluorofurans

Although methods for the synthesis of fluorofurans (compounds with the C_fur_-F bond) and fluoroalkylated furans have been developed, these methodologies often rely on non-furanic cyclic or acyclic fluorinated building blocks [93,94,109,110,111,112,113,114]. This approach has gained popularity due to its convenience and selective formation of target fluorinated molecules [57]. However, many of these methods require multi-step procedures for synthesizing specific substrates or involve costly reagents, which limits their synthetic applicability [57].

Preparation of representative fluorofurans through direct fluorination of furfural-derived substrates is summarized in Table 1. Nucleophilic fluorination via reactions with gaseous fluorine is not suitable for furan fluorination [94]. Nevertheless, some α-fluorofurans have been obtained from electron-poor furfural-derived substrates using nucleophilic fluorodenitration [78], through fluorodecarboxylation using the electrophilic fluorinating agent Selectfluor [115,116], or by employing rhodium-catalyzed heteroaryl exchange (Table 1) [117,118]. Another potential approach is the metalation-fluorination strategy, which has been effectively utilized for fluorination at both the α- and β-positions of the furan ring [119,120,121].

### 2.2. Synthesis of Fluoroalkylfurans

**Scheme 3:**
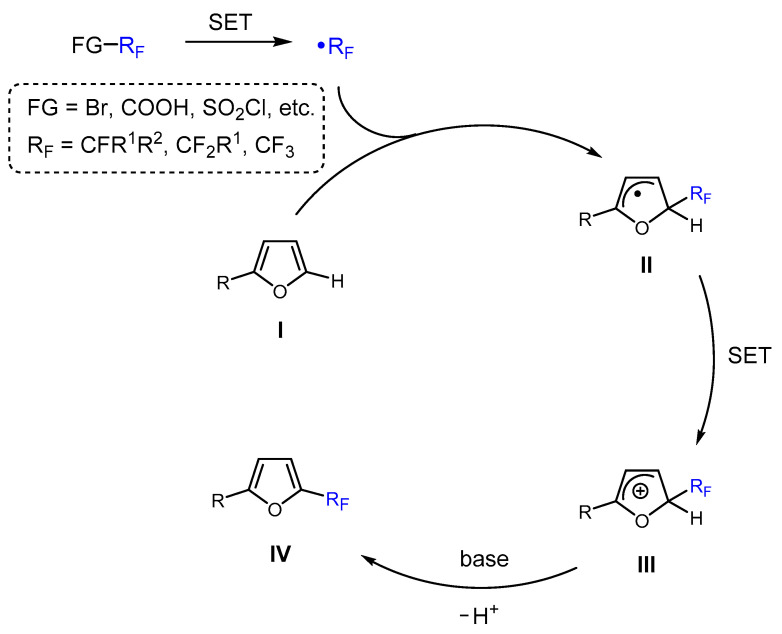
The general proposed mechanism for the radical fluoroalkylation of 2-substituted furans.

The recent methods for fluoroalkylation reactions involving furfural-derived furans can be classified into two main categories based on the mechanisms of generation and reactivity of fluoroalkyl species: (1) radical fluoroalkylation and (2) transition metal-catalyzed (cross)coupling. The mechanism of radical fluoroalkylation typically consists of three key stages (Scheme 3). The first stage involves the generation of the fluoroalkyl radical (•R_F_), which is a strong electrophile that readily reacts with electron-rich substrates such as furans **I**. These radicals can be produced from various fluorinated reagents, including halogenated fluoro-sources, fluorinated acids, anhydrides, sulfonyl chlorides, or hypervalent iodine reagents, typically through single electron transfer (SET) oxidation. In the subsequent stage, the •R_F_ radical inserts itself into the C5 position of the 2-substituted furanic core, resulting in the formation of a fluoroalkylated furanic radical **II**. Finally, the rearomatization of the furan ring leads to the desired 5-fluoroalkylated furans **IV**. This process generally involves a one-electron oxidation of **II** into the corresponding furanic cation **III** via an SET process, followed by deprotonation (Scheme 3). Various strategies have been employed for the radical fluoroalkylation of furans, including visible-light-mediated photocatalysis and photoredox catalysis, transition metal catalysis, and alternating current electrolysis (Table 2, Table 3, Table 4, Table 5 and Table 6).

Among the various methods for trifluoromethylation of furans, visible light-mediated radical trifluoromethylation, with or without photoredox catalysis, has recently garnered the most attention (Table 2 and Table 3) [92,122]. Photoredox-catalyzed trifluoromethylation, achieved through the generation of the electrophilic radical •CF_3_ using trifluoroacetic acid [123], trifluoroacetic anhydride (TFAA) [124,125], perfluoroarene iodine(III) trifluoroacetate [105], or trifluoromethanesulfonic anhydride [43] as CF_3_ sources has demonstrated high efficiency for the trifluoromethylation of acceptor-substituted furans such as 2-acetylfuran and 2-furoic acid derivatives (Table 2). Furfural-derived furans containing electron-donor substituents at the C2 position were trifluoromethylated through the transition-metal-mediated generation of trifluoromethyl radicals, with [38,39,106,126] or without [127,128,129] photoredox catalysis (Table 3). Zhong et al. reported a method for trifluoromethylation using perfluorocarboxylic anhydrides under metal-free conditions in the presence of urea and hydrogen peroxide [3]. This approach produced two α-trifluoromethylated furanic esters in good yields (Table 3, entries 9, 10).

**Table 2:** Radical trifluoromethylation of electron-poor furfural-derived furans.

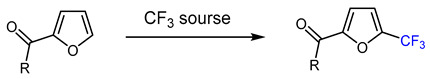				
**№**	**R**	**Reaction Conditions**	**Yield**	**Ref.**
1	Me	CF_3_COOH, (4-ClPh)_2_SO (2 eq.), Ru(bpy)_3_Cl_2_ (1 mol. %), DCE, RT, 427 nm LED	62	[123]
2	Me	TFAA, Me_2_C=NOH (2 eq.), Ru(bpy)_3_Cl_2_, DCE, RT, 390 nm LED	62	[125]
3	OMe	CF_3_COOH, (4-ClPh)_2_SO (2 eq.), Ru(bpy)_3_Cl_2_ (1 mol. %), DCE, RT, 427 nm LED	59	[123]
4	OMe	TFAA, Me_2_C=NOH (2 eq.), Ru(bpy)_3_Cl_2_, DCE, RT, 390 nm LED	74	[125]
5	OMe	TFAA, Ir(ppy)_3_ (3 mol. %), EtOAc, 40 °C, 30 W blue LED	64	[124]
6	OMe	C_6_F_5_I(OCOCF_3_)_2_, Ru(bpy)_3_(PF_6_)_2_ (2 mol. %), CH_3_CN, 35 °C, blue LED	41	[105]
7	OMe	(CF_3_SO_2_)_2_O, Ru(bpy)_3_Cl_2_ (2 mol. %), pyridine, DCE, RT, LED	74	[43]
8	OMe	CF_3_SO_2_Na, [Ru(bpy)_3_][PF_6_]_2_, LiClO_4_, CH_3_CN, RT, blue LED, CCE at 4.0 mA	77	[130]
9	OMe	CF_3_SO_2_Cl, K_2_HPO_4_, 100 Hz, (+)C/(−)C, 4.8 V, CH_3_CN, 0.125 M LiClO_4_	32	[131]
10	OC_8_H_17_	CF_3_COOH, (4-ClPh)_2_SO (2 eq.), Ru(bpy)_3_Cl_2_ (1 mol. %), DCE, RT, 427 nm LED	58	[123]
11	OC_8_H_17_	TFAA, Me_2_C=NOH (2 eq.), Ru(bpy)_3_Cl_2_, DCE, RT, 390 nm LED	60	[125]
12	NHMe	CF_3_SO_2_Na, [Ru(bpy)_3_][PF_6_]_2_, LiClO_4_, CH_3_CN, RT, blue LED, CCE at 4.0 mA	69	[130]
13	NH(CH_2_COOEt)	CF_3_SO_2_Na, [Ru(bpy)_3_][PF_6_]_2_, LiClO_4_, CH_3_CN, RT, blue LED, CCE at 4.0 mA	70	[130]
14	OH	TFAA, Me_2_C=NOH (2 eq.), Ru(bpy)_3_Cl_2_, DCE, RT, 390 nm LED	68	[125]
15	OH	(CF_3_SO_2_)_2_O, Ru(bpy)_3_Cl_2_ (2 mol. %), pyridine, DCE, RT, LED	72	[43]

**Table 3:** Radical trifluoromethylation of electron-rich furfural-derived furans.

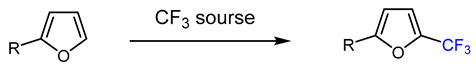				
**№**	**R**	**Reaction Conditions**	**Yield**	**Ref.**
1	CH_3_	CF_3_SO_2_Cl, Ru-cat., Mg acetate, CH_3_CN, RT, blue light	86	[106]
2	CH_3_	CF_3_SO_2_Cl, Ru(phen)_3_Cl_2_ (1 mol. %), K_2_HPO_4_, CH_3_CN, RT, 26 W light	87	[38]
3	CH_3_	Co(III)-CF_3_ complex, NMP, RT, 16 W light	87	[127]
4	CH_3_	CF_3_I, Ru(bpy)_3_Cl_2_ (1 mol. %), TMEDA, CH_3_CN, blue LED	75	[36]
5	CH_3_	TMSCF_3_ (2eq.), PhI(OAc) (2 eq.), AgF (25 mol. %), DMSO, RT	51	[128]
6	CH_3_	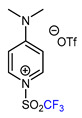 , Ir(ppy)_3_ (2 mol.%), KBr, CH_2_Cl_2_, RT, blue LED	63	[132]
7	CH_3_	CF_3_SO_2_Cl (2 eq.), K_2_HPO_4_, 100 Hz, (+)C/(−)C, CH_3_CN, 0.125 M LiClO_4_	43	[131]
8	CH_2_S(CHO)	CF_3_SO_2_Cl, CdSe (10 mol. %), K_2_HPO_4_, CH_3_CN, RT, blue LED	68	[39]
9	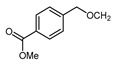	TFAA, urea-hydrogen peroxide, CH_2_Cl_2_, 0 °C	49	[3]
10	CH_2_OBn	(C_2_F_5_CO)_2_O, urea-hydrogen peroxide, CH_2_Cl_2_, 0 °C	46	[3]
11	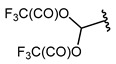	CF_3_COOH, XeF_2_, CH_2_Cl_2_, 0 → 20 °C, then H_2_O, CF_3_COOH	30 ^1^	[133]

^1^ Yield of 5-(trifluoromethyl)-2-furaldehyde.

Mono-, di-, and perfluoroalkyl-functionalized heterocycles are less common than aryl-fluorinated (Ar-F) or aryl-trifluoromethylated (Ar-CF_3_) compounds, but they remain of interest due to their biological activity and applications in materials science [134,135]. Representative and efficient methods for fluoroalkylation of both electron-poor (Table 4 and Table 5) and electron-rich (Table 6) furans are outlined below. The nucleophilic fluorinating reagent Deoxo-Fluor exhibited significant selectivity in the deoxyfluorination of 5-nitro-2-furaldehyde to produce 2-(difluoromethyl)-5-nitrofuran (Table 5, entry 8), as well as in the fluorination of several 2,5-disubstituted furanic aldehydes [136]. Additionally, Zhang et al. utilized another nucleophilic fluorinating reagent DAST for the direct difluoromethylation of furfuryl acetate at the C5 position (Table 6, entry 13) [137].

**Table 4:** Representative and most efficient methods of introduction of CF-, CF_2_- and perfluoroalkyl groups to alkyl 2-furoates.

№	Furan	Reaction Conditions	Product	Yield	Ref.
1	Methyl 2-furoate	ICF_2_SO_2_F, [Ir(dFCF_3_ppy)_2_(bpy)]PF_6_ (1 mol. %), AgOTf (1 eq.), DMC, RT, blue LED	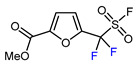	80	[138]
2	Methyl 2-furoate	BrCF_2_SO_2_(4-Cl-Ph), Mn_2_(CO)_10_ (10 mol. %), Davephos (15 mol. %), K_2_CO_3_, DCM, white LED	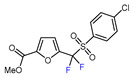	86	[139]
3	Methyl 2-furoate	BrCF_2_CO_2_Et, Pd(PPh_3_)_4_ (5 mol. %), Xantphos (10 mol. %), K_2_CO_3_, dioxane, Ar, 110 °C	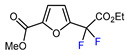	67	[140]
4	Methyl 2-furoate	C_3_F_7_,XeF_2_, CH_2_Cl_2_, RT	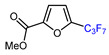	33	[141]
5	Methyl 2-furoate	IC_4_F_9_ (30 eq.), CuI (10 mol. %), phen (20 mol. %), 2,4,6-collidine, CH_2_Cl_2_, 110 °C	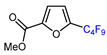	46	[142]
6	Methyl 2-furoate	IC_4_F_9_, [Cu(bcp)DPEphos]PF_6_ (0.1 eq.), KOAc, CH_2_Cl_2_, blue LED	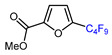	51	[143]
7	Methyl 2-furoate	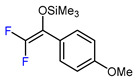 , Cu(OTf)_2_, CH_3_CN-H_2_O, 0 °C	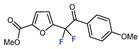	61	[144]
8	Methyl 2-furoate	BrCF(CO_2_Et)_2_, *fac-*[Ir(ppy)_3_] (1mol. %), K_2_CO_3_, DMF, RT blue, LED	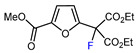	72	[145]
9	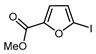	[Ph_4_P]^+^[Cu(CF_2_H)_2_]^–^ (1 eq.), DMAc, 90 °C	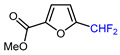	56	[146]
10	Ethyl 2-furoate	BrCF_2_CN, [Ir(dtbbpy)(ppy)_2_][PF_6_], DMF, blue LED	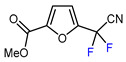	74	[147]
11	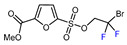	[Ru(bpy)_3_]Cl_2_ 6H_2_O (0.1 mol. %), NBu_3_ (1.5 eq.), HCOOH (1.5 eq.), DMSO, RT, blue LED	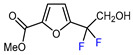	73	[148]
12	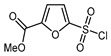	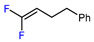 (1.75 eq.), Pd(OAc)_2_ (5 mol. %), SIPr•Cl (10 mol. %), CuCl, Li_2_CO_3_, dioxane, 120 °C	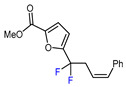	50	[149]

Transition metal-mediated C-H functionalization and radical visible light-mediated fluoroalkylation, with or without photoredox catalysis, demonstrated high efficiency for the introduction of fluoroalkyl groups into furans. Using these methods, mono-, di-, and perfluoroalkylated furanic building blocks were obtained with notable efficiency from electron-poor furans including 2-furoates (Table 4), furfural, 2-furoic acid, and other derivatives (Table 5) [14,140,150,151] and electron-rich furans such as 2-methylfuran, furfural-based dioxolane acetal, furfuryl alcohol and derivatives (Table 6). Coupling strategies involving furyl iodides [146,152,153] or sulfonyl chlorides [149] were also effectively employed for the fluorination of acceptor-substituted furans (Table 4, entries 9 and 12, and Table 5, entries 4–7).

**Table 5:** Representative and most efficient methods of introduction of CF-, CF_2_- and perfluoroalkyl groups to other electron-poor furfural-derived furans.

№	Furan	Reaction Conditions	Product	Yield	Ref.
1	Furfural	BrCF_2_CO_2_Et, CuI (10 mol. %), PMDETA (1.5 eq.), CH_3_CN, 80 °C	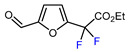	50	[154]
2	Furfural	BrCF_2_CO_2_Et, Ir(ppy)_3_ (0.5 mol. %), phen (20 mol. %), NaOAc (2 eq.), CH_3_CN, RT, 3 W blue LED	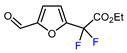	44	[155]
3	Furfural	BrCF_2_CO_2_Et, [Ru(*p*-cymene)Cl_2_]_2_ (5 mol. %), Na_2_CO_3_, AgTFA (2 eq.), *N*-Ac-*L*-Iso (30 mol. %), *tert*-butylamine (0.5 eq.), DCE, 155 °C	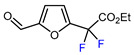	62	[156]
4	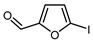	TMSCF_2_Br (7.2 eq.), CuBr (5.2 eq.), ArSH (12 eq.), phen, 18-crown-6, NaH, C_6_F_6_, NMP, 60 °C	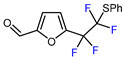	81	[153]
5	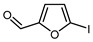	TMSCF_3_ (1.8 eq.), C_6_F_5_TMS, CuCl (5.2 eq.), KF (12 eq.), 60 °C	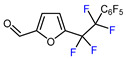	75	[152]
6	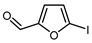	(DMPU)_2_Zn(CHF_2_)_2_, (dppf)Ni(COD) (15 mol. %), DMSO, RT	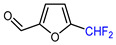	78	[157]
7	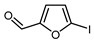	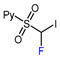 , Et_2_Zn (0.6 eq.), CuI (1.3 eq.), DMF, RT	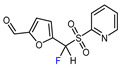	67	[158]
8	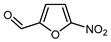	Deoxo-Fluor (1.5 eq.) CH_2_Cl_2_, RT	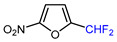	68	[136]
9	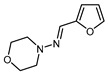	BrCF_2_CO_2_Et, CuBr_2_ (10 mol. %), DTBDPy (10 mol. %), B_2_pin_2_ (30 mol. %), NaHCO_3_, dioxane, 80 °C	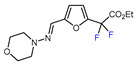	51	[159]
10	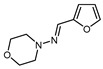	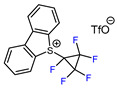 (0.33 eq.), NaHCO_3_, CH_3_CN, RT, 462 nm LED	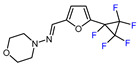	69	[160]
11	2-Acetylfuran	BrCF_2_CO_2_Et, CuI (10 mol. %), PMDETA (1.5 eq.), CH_3_CN, 80 °C	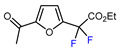	53	[154]
12	2-Acetylfuran	BrCF(CO_2_Et)_2_, *fac-*[Ir(ppy)_3_] (1mol. %), K_2_CO_3_, DMF, RT, blue LED	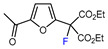	65	[145]
13	2-Furonitrile	ICF_2_SO_2_F, [Ir(dFCF_3_ppy)_2_(bpy)]PF_6_ (1 mol. %), AgOTf (1 eq.), DMC, RT, blue LED	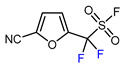	85	[138]

As shown in Table 2, Table 3, Table 4, Table 5 and Table 6, most fluoroalkylation methods were tested on either C2 donor- or acceptor-substituted furanic substrates. Some methodologies, such as Pd-catalyzed difluoromethylation using ethyl 2-bromo-2,2-difluoroacetate [140], Cu-catalyzed perfluoroalkylation with IC_4_F_9_ [142], and electrocatalytic trifluoromethylation [131] demonstrated considerable efficiency for both donor- and acceptor-substituted furans. However, the applicability of many reported fluoroalkylation systems of furans is not fully established, as the range of tested furanic substrates was often limited to highly stable or non-functionalized compounds like 2-methylfuran [14,36,106,126,127,129,134,161,162], methyl 2-furoate [146,148], or 2-acetylfuran [151]. In some cases, fluoroalkylation resulted in significantly lower yields compared to other heteroaromatic substrates, or even failed [22,163,164,165].

**Table 6:** Representative and most efficient methods of introduction of CF-, CF_2_- and perfluoroalkyl groups to electron-rich furfural-derived furans.

№	Furan	Reaction Conditions	Product	Yield	Ref.
1	2-Methylfuran	BrCF_2_CO_2_Et, CuI (10 mol. %), K_2_CO_3_, DMF, 60 °C	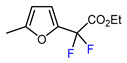	61	[150]
2	2-Methylfuran	BrCF_2_CO_2_Et, Pd(PPh_3_)_4_ (5 mol. %), Xantphos (10 mol. %), K_2_CO_3_, dioxane, Ar, 110 °C	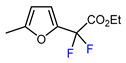	57	[140]
3	2-Ethylfuran	BrCF_2_CO_2_Et, *fac-*[Ir(ppy)_3_], K_3_PO_4_, DMF, RT, 7 W blue LED	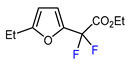	83	[166]
4	2-Ethylfuran	PhSO_2_CF_2_I, Pd_2_(dba)_3_ (5 mol. %), Xantphos (20 mol. %), Cs_2_CO_3_, CHCl_3_, 60 °C	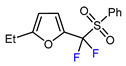	96	[167]
5	2-Ethylfuran	PhSO_2_CF_2_I, Ru(bpy)_3_Cl_2_•6H_2_O (1 mol. %), K_2_HPO_4_, CH_2_Cl_2_, 40 °C, 26W light	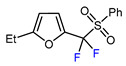	96	[168]
6	2-Ethylfuran	BrCF_2_CO_2_Et, Pd(PPh_3_)_4_ (5 mol. %), Xantphos (10 mol. %), K_2_CO_3_, dioxane, Ar, 110 °C	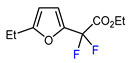	63	[140]
7	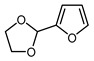	PhSO_2_CF_2_I, Ru(bpy)_3_Cl_2_•6H_2_O (1 mol. %), K_2_HPO_4_, CH_2_Cl_2_, 26 W light, 40 °C	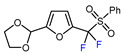	90	[168]
8	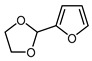	BrCF_2_CO_2_Et, Pd(PPh_3_)_4_ (5 mol. %), Xantphos (10 mol. %), K_2_CO_3_, dioxane, Ar, 110 °C	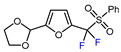	80	[167]
9	Furfuryl alcohol	IC_4_F_9_ (30 eq.), CuI (10 mol. %), 1,10 phenantroline (20 mol. %), 2,4,6-collidine, CH_2_Cl_2_, 110 °C	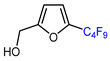	74	[142]
10	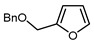	BrCF_2_CO_2_Et, CuI (10 mol. %), K_2_CO_3_, DMF, 80 °C	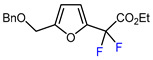	64	[150]
11	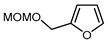	BrCF_2_CO_2_Et, CuI (10 mol. %), K_2_CO_3_, DMF, 80 °C	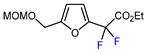	65	[150]
12	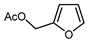	BrCF_2_CO_2_Et, CuI (10 mol. %), K_2_CO_3_, DMF, 80 °C	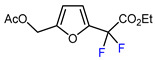	63	[150]
13	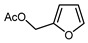	DAST (3.5 eq.), CH_2_Cl_2_, RT	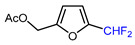	60	[137]

Synthesis of β-fluorinated and β-fluoroalkylated furans through direct fluoroalkylation presents challenges; however, it can be achieved using fluorinated acyclic precursors [70,169,170,171,172,173]. On the other hand, the dienic nature of the furan ring offers the construction of fluorofurans via Diels–Alder chemistry. Regioselective hydrogenation of the unsubstituted double bond in Diels–Alder adducts **7** and **8** formed from electron-rich furans and hexafluorobutyne, followed by temperature-induced elimination of ethylene, resulted in β,β-bis-trifluoromethylated furans **9** and **10** in good overall yields (Table 7) [174].

In the same study, bis-trifluoromethylated furans **11**–**15** containing electron-accepting functional groups at the C2 position were synthesized in a single step through high-temperature reactions with heptafluorobutene (Table 7). This alkene served as a strong dienophile reacting with 2-furonitrile, 2-furoic acid or its esters, and even with furfural, producing the desired fluorinated trisubstituted furans in good yields [174].

## 3. Application of Furfural-Derived Fluorofurans as Building Blocks in Drug Development

**Scheme 4:**
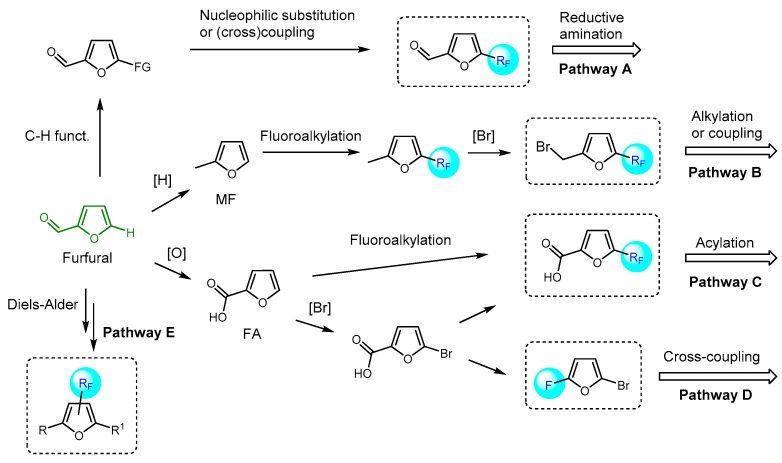
Representative reaction map for the synthesis of fluorinated and fluoroalkylated furanic building blocks for drug development (possible structures of these building blocks are enclosed in dashed squares).

Introduction of fluorinated furan derivatives into biologically active scaffolds has garnered significant attention [62,63]. The polyfunctional nature of furfural-derived furans, which allow several reactivity patterns such as the modification of the substituents at the C2 position, the functionalization of the C5-H bond, and the dienic reactivity of the furan ring, provides efficient and diverse chemical access to various fluorofuran or fluoroalkylfuran derivatives. A possible reaction map for the synthesis of fluorinated furanic building blocks starting from furfural is presented in Scheme 4. The introduction of fluorinated furan moieties into biologically active scaffolds can be achieved through reductive amination with fluorinated furanic aldehydes (pathway A). The 2-(bromomethyl)-5-(fluoroalkyl)furans, obtainable through the bromination of corresponding 2-(methyl)-5-(fluoroalkyl)furans, can serve as an alkylating agent or substrate in various coupling reactions (pathway B) [175,176,177,178,179]. C5-fluorinated furoic acids can also act as convenient building blocks for the introduction of fluorofuran or fluoroalkylfuran groups via acylation (pathway C). Another potential approach involves the use of halogenated or sulfonated α-fluorofurans in cross-coupling reactions (pathway D). Functionalized furans containing fluorinated functional groups at the β-positions may be potentially synthesized using the Diels–Alder strategy (pathway E). Recent and representative examples of the application of fluorofuran and fluoroalkylfuran reagents in drug development using these approaches are discussed below.

**Scheme 5:**
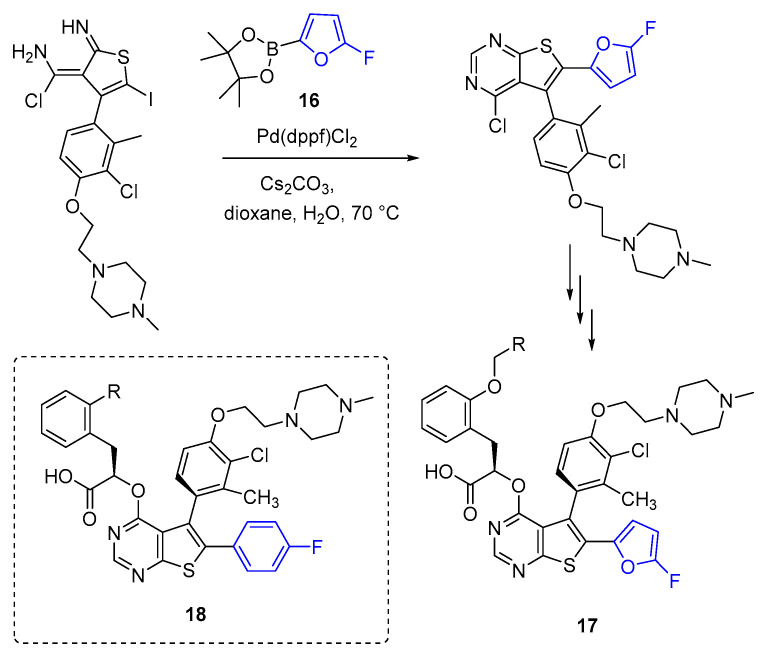
Application of fluorofuranic pinacol ester **16** in the synthesis of MCL1 inhibitors. Here and below, blue color indicates the fluorine-functionalized furan or other fluorinated fragment.

α-Fluorofuran **16** with a boronic acid pinacol ester group can be synthesized from 5-bromo-2-furoic acid through decarbonylative fluorination, followed by a reaction with pinacol diborate [180]. Cross-coupling of **16** was employed to introduce the 2-fluorofuran fragment into targeted molecules in the development of inhibitors of MCL1 for cancer therapy (Scheme 5) [65,181]. Compound **2** (Table 1) binds to the BH3-binding groove of MCL1 with high selectively and affinity [65]. In later studies, a number of similar 4-fluorofur-2-yl (**17**) and 4-fluorophenyl (**18**) derivatives were discovered. Despite the lower activity of 4-fluorophenyl derivatives **18** in vitro, a fluorophenyl analogue demonstrated the in vivoefficacy comparable to that of fluorofuryl derivatives **17**, prompting further research on fluorophenyl derivatives **18** [181].

**Scheme 6:**
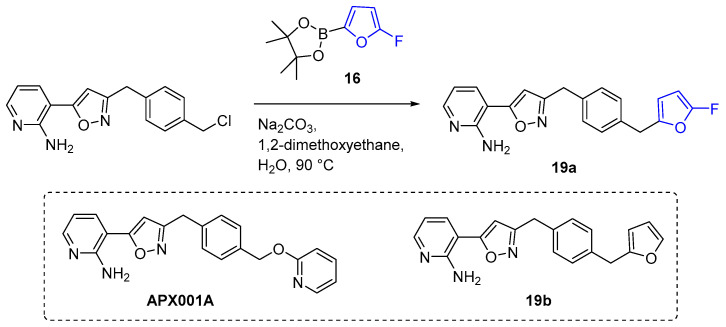
Synthesis of α-fluorofuryl analogue of manogepix.

Pinacol boronic ester **16** was also utilized in the synthesis of compound **19a**, which is a α-fluorofuran analogue of manogepix (APX001A), the active moiety of a novel experimental antifungal drug fosmanogepix (Scheme 6). The antifungal activity of **19a** was evaluated against *Cryptococcus neoformans* and *Cryptococcus gattii* [182]. Compound **19a** exhibited notably lower MIC values and a longer half-life compared to manogepix and their furanic nonfluorinated analogue **19b**. Therefore, it can be concluded that the incorporation of a fluorine atom into the furan fragment of the manogepix analogue **19b** enhances its metabolic stability.

**Scheme 7:**
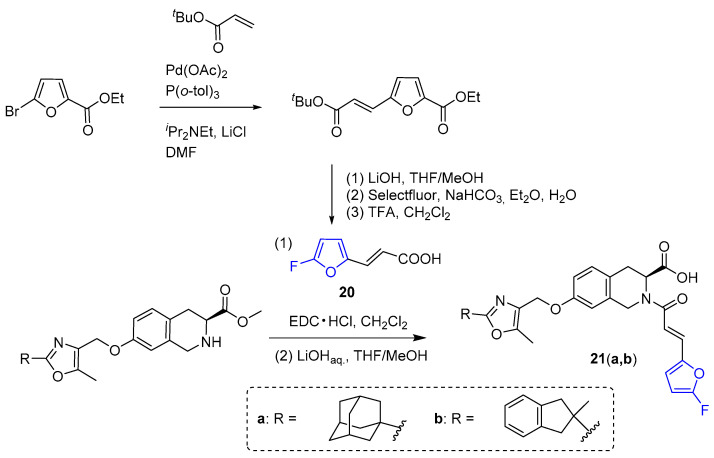
Synthesis of acrylic 3-(5-fluorofuryl)-substituted 1,2,3,4-tetrahydroisoquinoline-3-carboxylic acids using 3-(5-fluorofuryl)-acrylic acid **20**.

Various 1,2,3,4-tetrahydroisoquinoline-3-carboxylic acids, including 3-(5-fluorofuryl)-substituted derivatives **21**(**a,b**), were synthesized and evaluated for their ability to partially activate peroxisome proliferator-activated receptor gamma (PPARγ), which plays a key role in lowering the blood sugar level in patients with type 2 diabetes [183]. In this series, compound **21b** containing an indanyl group exhibited reduced affinity for the PPARγ receptor in comparison to its non-fluorinated counterpart that was selected as the lead compound for further studies. The compounds **21**(**a,b**) were synthesized from 3-(5-fluorofuryl)acrylic acid **20**, which was obtained from ethyl 5-bromofuran-2-carboxylate through decarbonylative fluorination using Selectfluor (Scheme 7) [183].

**Scheme 8:**
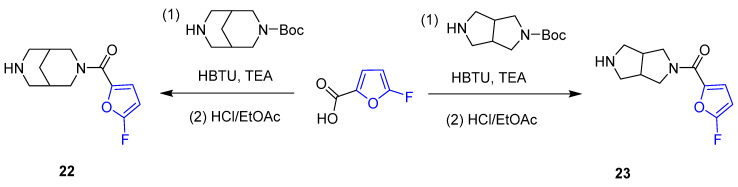
Application of 5-fluorofuranic acid for the synthesis of amides **22** and **23**.

5-Fluorofuranic amides **22** and **23** were synthesized with the aim of searching for new effective selective agonists of *α4β2* nicotinic acetylcholine receptors (nAChR) [184]. These amides were obtained through the acylation of corresponding Boc-protected amines with 5-fluoro-2-furoic acid using HBTU as a carboxylic group activator, followed by Boc deprotection (Scheme 8) [184]. SAR studies indicated that furoamide **22** exhibited sufficiently high efficacy as an *α*4*β*2 nAChR-selective agonist among other studied heterocyclic amides. However, the significant undesirable activation of nAChR receptors specifically located in the ganglia hindered the potential of 5-fluoro-2-furyl derivatives **22** and **23** as lead compounds for further in vivotesting.

**Scheme 9:**
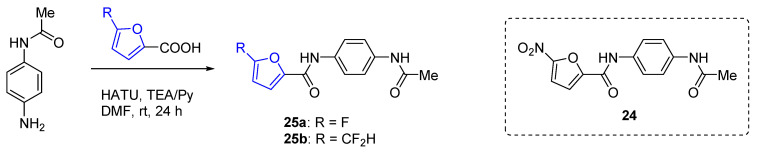
Synthesis of amides **25**(**a**,**b**) by acylation with C5-fluorinated furoic acids.

To mitigate potential genotoxicity associated with the formation of nitrofuran metabolites, the nitro group in furanic orally available UT inhibitor **24** was replaced with more stable fluorine or difluoromethyl group [185]. These new compounds **25**(**a,b**) were synthesized by condensing an *N*-(4-aminophenyl)acetamide with fluorinated furan-2-carboxylic acids in the presence of a base and HATU (Scheme 9). Unfortunately, the substitution of the nitro group with fluorine or difluoromethyl group resulted in a significant reduction in biological activity for this class of compounds.

**Scheme 10:**
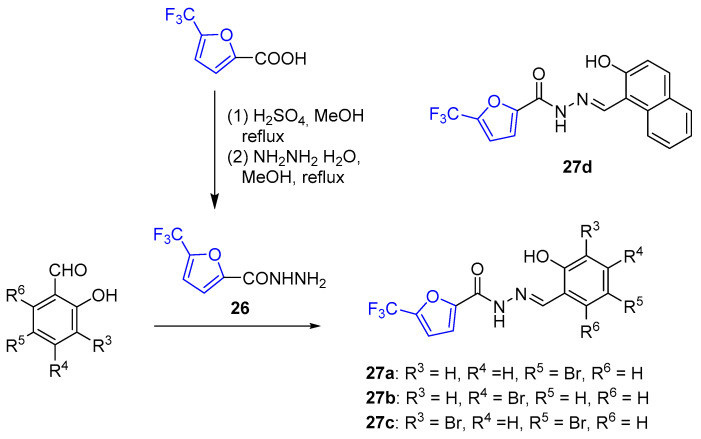
Synthesis of *α*-trifluoromethylated furanic hydrazides **27**(**a**–**c**).

The α-trifluoromethylfuran derivatives **27**(**a**–**d**) displayed significantly higher antifungal activity against *C. neoformans* compared to their non-fluorinated furanic analogs. However, these compounds were less potent than their thiophene counterparts. Although in subsequent tests on fungicidal or fungistatic activity, as well as on selectivity against fungi/human cells, none of trifluoromethylated furans were sufficiently active and selective to be included in the leader compound set, the results of these experiments indicate the importance of substituents in furan moieties for high antifungal activity [186]. The abovementioned derivatives **27**(**a**–**d**) were obtained from the corresponding 5-trifluoromethylfurane hydrazide **26**, which was synthesized starting from 2-trifluoromethylfuran-5-carboxylic acid (Scheme 10).

**Scheme 11:**
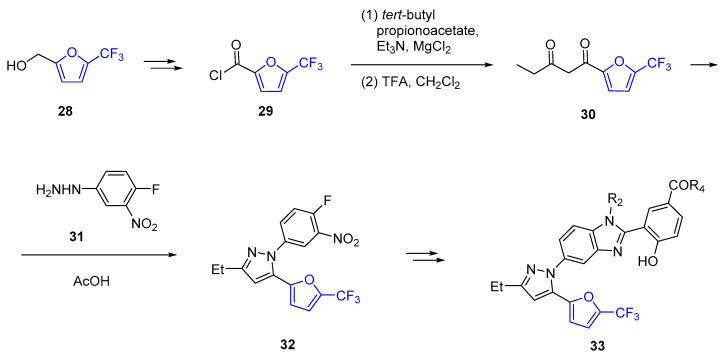
Synthesis of HIV-1 inhibitor **33** containing *α*-trifluoromethylated furan.

Using a series of 5-(5-furan-2-yl-pyrazol-1-yl)-1*H*-benzimidazole derivatives that inhibited the assembly of the HIV-1 capsid, the effect of site-selective modifications at N1, C2 and C16 of this scaffold was clarified. Replacing the methyl substituent at C16 with a trifluoromethyl group (compound **33**) produced a six-fold gain in the antiviral potency significantly affecting the IC_50_ values [187]. The key stage of the synthesis of these 5-(5-furan-2-ylpyrazol-1-yl)-1H-benzimidazoles was the acidic condensation of hydrazine **31** with fluorinated furanic diketone **30**, which in turn was obtained from 5-(trifluoromethyl)furan derivative **28** (Scheme 11).

**Scheme 12:**
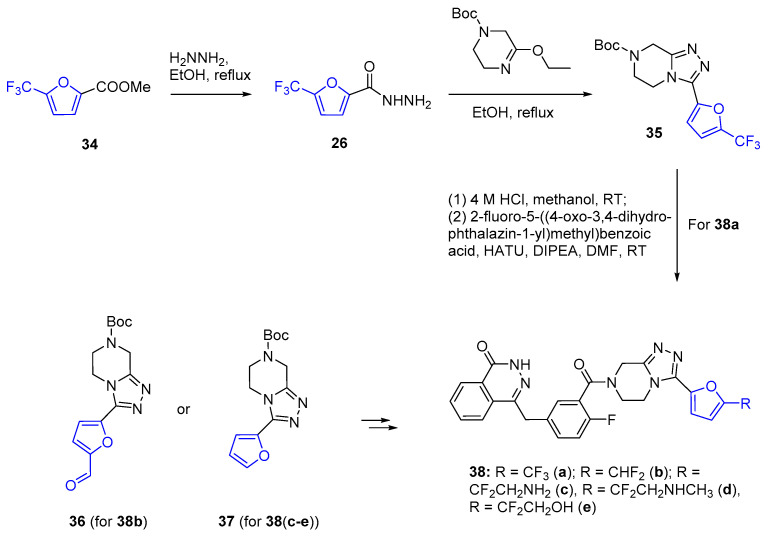
Synthesis of α-fluoroalkylated furanic phthalazin-1(2*H*)-ones **38**(**a**–**e**).

Phthalazin-1(2*H*)-ones with furan as a side substituent showed the highest levels of antiproliferative activities against olaparib- and talazoparib-resistant Capan-1 cells [61]. In attempts to find the optimal substituents at the α-carbon of the furan ring, derivatives **38**(**a**-**e**) containing the furan substituted at the α-carbon position by various fluoroalkyl moieties were obtained (Scheme 12).

The initial building block for the synthesis of **38a** was α-trifluoromethylated furanic ester **34**. This compound was converted into triazole **35** through a two-step process involving modification to hydrazide **26**, followed by cyclization. The α-difluoromethyl fragments in **38b** and **38**(**c-e**) were introduced via fluorination at α-position of the furanic ring in intermediates **36** and **37**, respectively. Among these compounds, water-soluble derivatives **38**(**c-e**) demonstrated improved inhibitory activity, but reduced potency towards drug-resistant cells (olaparib- and talazoparib-resistant Capan-1 cells) [61]. Thus, unfortunately, none of these fluorinated furan structures emerged as the leader compound in this study.

## 4. Conclusions

Although numerous methods have been reported for introducing fluorine or fluorine-containing functional groups into various aromatic and heteroaromatic compounds, direct functionalization approaches for furfural-derived furans remain underdeveloped. Current methodologies for synthesizing α-fluorofurans and α-fluoroalkylfurans still suffer from low yields, limited generality, or reliance on an expensive reagent. Furthermore, furans containing fluorinated functional groups at the β-positions were synthesized from furfural-derived substrates only through indirect methods. The existing fluoroalkylation techniques were typically tested on a limited range of relatively stable furfural-derived furan substrates, such as 2-methylfuran, methyl 2-furoate, and 2-acetylfuran.

Fluorinated and fluoroalkylated furans, benefiting from the unique properties of both furan and fluorine functionalities, hold significant promise for drug design. However, none of these compounds have yet been approved for clinical use by the Food and Drug Administration (FDA). On the other hand, fluorination significantly increases the exothermic effect of Diels–Alder reactions and lowers the activation energy barriers, which is more pronounced at the α-position of furan compared to the β-position [188,189]. Consequently, incorporating fluorofurans or fluoroalkylfurans into the structures of Diels–Alder-based dynamers could substantially influence their thermal and chemical stability. However, our literature review revealed no reported examples of fluorinated furans being applied in the development of Diels–Alder dynamers.

To achieve more sustainable fluorinated polymers and pharmaceuticals, it is essential to develop more versatile and efficient methods for introducing fluorinated functional groups into furan derivatives. Key considerations include the applicability of these methods to a range of furfural-derived donor- or acceptor-substituted furan substrates, including those containing sensitive functional groups. Establishing such methodologies would not only expand synthetic possibilities but also promote the active and efficient utilization of plant biomass in drug design and materials science through the development of fluoro-functionalized furans.

## Figures and Tables

**Table 1:** Synthesis of fluorofurans from furfural-derived furanic building blocks.

№	Furanic Substrate	Reaction Conditions	Product	Yield (%)	Ref.
1	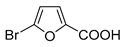	Selectfluor (1 eq.), CCl_4_/sat. NaHCO_3_ (2 eq.)	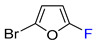	27	[115]
2	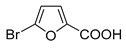	Selectfluor (2 eq.), KF (4 eq.), DCE/H_2_O (2:1), 70 °C	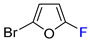	57	[116]
3	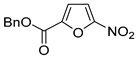	(1) KF, PPh_4_Br, sulfolane, 140 °C; (2) Pd/C, H_2_ (1 atm.), MeOH, RT	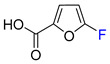	56	[78]
4	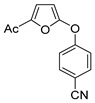	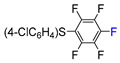 (4 eq.), RhH(dppBz)_2_ (10 mol. %), PhCl, reflux	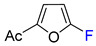	53	[118]

**Table 7:** Indirect synthesis of β,β-bis-trifluoromethylated furans using Diels–Alder chemistry [174].

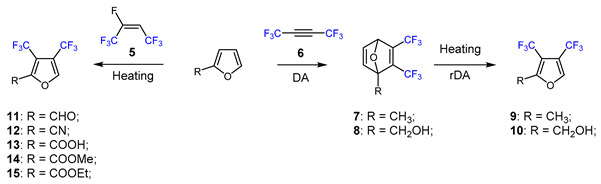				
**№**	**Substrate**	**Reaction Conditions**	**Product**	**Yield**
1	2-Methylfuran	**6** (1 eq.), THF, rocking furnace, 130 °C, 24 h	**7**	86
2	Furfural alcohol	**6**, THF, rocking furnace, 130 °C, 24 h	**8**	92
3	**7**	H_2_, Pt/C, THF, then 400 °C	**9**	89
4	**8**	H_2_, Pt/C, THF, then 400 °C	**10**	87
5	Furfural	**5** (1.6 eq.), quartz tube, 225 °C, 24 h	**11**	51
6	2-Furonitrile	**5** (1.5 eq.), quartz tube, 250 °C, 24 h	**12**	71 + 21 ^1^
7	2-Furoic acid	**5** (1.5 eq.), quartz tube, 300 °C, 24 h	**13**	39 ^1^
8	Methyl 2-furoate	**5** (1.4 eq.), quartz tube, 250 °C, 24 h	**14**	85
9	Ethyl 2-furoate	**5** (1.5 eq.), quartz tube, 250 °C, 24 h	**15**	89

^1^ Yield of 3,4-bis(trifluoromethy1)furan.

## Data Availability

Not applicable.

## Abbreviations

The following abbreviations are used in this manuscript:

AIDS	Acquired immunodeficiency syndrome
BH3	B-cell lymphoma homology 3
bpy	2,2′-Bipyridine
CAN	Covalent adaptable network
CCE	Constant current electrolysis
COD	1,3-Cyclooctadiene
DA	Diels–Alder
DAST	Diethylaminosulfur trifluoride
dba	Dibenzylideneacetone
DCE	1,2-Dichloroethane
Deoxo-Fluor	Bis(2-methoxyethyl)aminosulfur trifluoride
DIPEA	N,N-Diisopropylethylamine
DMC	Dimethyl carbonate
DMF	N,N-Dimethylformamide
DMPU	1,3-Dimethyl-3,4,5,6-tetrahydro-2-pyrimidinone
DMSO	Dimethyl sulfoxide
dppBz	1,2-Bis(diphenylphosphino)benzene
dppf	1,1′-Bis(diphenylphosphino)ferrocene
DTBDPy	4,4′-Di-*tert*-butyl-2,2′-dipyridyl
EDC	1-Ethyl-3-(3-dimethylaminopropyl)carbodiimide
FDA	Food and Drug Administration
FMDA	Furan–maleimide Diels–Alder
HATU	Hexafluorophosphate azabenzotriazole tetramethyl uronium
HBTU	O-(benzotriazol-1-yl)-N,N,N,N-tetramethyluronium hexafluorophosphate
HIV	Human immunodeficiency virus
IC_50_	Half maximal inhibitory concentration
LED	Light-emitting diode
MCL1	Myeloid cell leukemia 1
MIC	Minimum inhibitory concentration
MOM	Methoxymethyl
nAChR	α4β2 Nicotinic acetylcholine receptors
NMP	N-Methyl-2-pyrrolidone
phen	1,10-Phenanthroline
PMDETA	N,N,N′,N″,N″-Pentamethyldiethylenetriamine
PPARγ	Peroxisome proliferator-activated receptor gamma
ppy	2-Phenylpyridine
rDA	Retro Diels–Alder
RT	Room temperature
SAR	Structure—activity relationship
SelectFluor	1-(Chloromethyl)-4-fluoro-1,4-diazabicyclo [2.2.2]octane-1,4-diium ditetrafluoroborate
SET	Single electron transfer
SIPr•Cl	1,3-Bis [2,6-bis(1-methylethyl) phenyl]-1H-imidazolium chloride
TEA	Triethylamine
TFA	Trifluoroacetic acid
TFAA	Trifluoroacetic anhydride
THF	Tetrahydrofuran
TMEDA	Tetramethylethylenediamine
